# Probe-Dependent Negative Allosteric Modulators of the Long-Chain Free Fatty Acid Receptor FFA4

**DOI:** 10.1124/mol.116.107821

**Published:** 2017-06

**Authors:** Kenneth R. Watterson, Steffen V. F. Hansen, Brian D. Hudson, Elisa Alvarez-Curto, Sheikh Zahir Raihan, Carlos M. G. Azevedo, Gabriel Martin, Julia Dunlop, Stephen J. Yarwood, Trond Ulven, Graeme Milligan

**Affiliations:** Centre for Translational Pharmacology, Institute of Molecular, Cell and Systems Biology, College of Medical, Veterinary and Life Sciences, University of Glasgow, Glasgow, United Kingdom (K.R.W., B.D.H., E.A.-C., S.Z.R., J.D., S.J.Y., G.M.); Department of Physics, Chemistry and Pharmacy, University of Southern Denmark, Odense, Denmark (S.V.F.H., C.M.G.A., G.M., T.U.); and Institute of Biological Chemistry, Biophysics and Bioengineering, School of Engineering and Physical Sciences, Heriot-Watt University, Edinburgh, United Kingdom (S.J.Y.)

## Abstract

High-affinity and selective antagonists that are able to block the actions of both endogenous and synthetic agonists of G protein–coupled receptors are integral to analysis of receptor function and to support suggestions of therapeutic potential. Although there is great interest in the potential of free fatty acid receptor 4 (FFA4) as a novel therapeutic target for the treatment of type II diabetes, the broad distribution pattern of this receptor suggests it may play a range of roles beyond glucose homeostasis in different cells and tissues. To date, a single molecule, 4-methyl-*N*-9*H*-xanthen-9-yl-benzenesulfonamide (AH-7614), has been described as an FFA4 antagonist; however, its mechanism of antagonism remains unknown. We synthesized AH-7614 and a chemical derivative and demonstrated these to be negative allosteric modulators (NAMs) of FFA4. Although these NAMs did inhibit FFA4 signaling induced by a range of endogenous and synthetic agonists, clear agonist probe dependence in the nature of allosteric modulation was apparent. Although AH-7614 did not antagonize the second long-chain free fatty acid receptor, free fatty acid receptor 1, the simple chemical structure of AH-7614 containing features found in many anticancer drugs suggests that a novel close chemical analog of AH-7614 devoid of FFA4 activity, 4-methyl-*N*-(9*H*-xanthen-9-yl)benzamide (TUG-1387), will also provide a useful control compound for future studies assessing FFA4 function. Using TUG-1387 alongside AH-7614, we show that endogenous activation of FFA4 expressed by murine C3H10T1/2 mesenchymal stem cells is required for induced differentiation of these cells toward a more mature, adipocyte-like phenotype.

## Introduction

Free fatty acid receptor 4 (FFA4) (Davenport et al., 2013; Milligan et al., 2017), which is also still frequently designated as GPR120 (Hirasawa et al., 2005), was identified in 2005 as a G protein–coupled receptor (GPCR) responsive to long-chain free fatty acids (Hirasawa et al., 2005). Since then, not least because essential and other polyunsaturated *ω*-3 fatty acids with health-promoting properties are among the most potent fatty acid activators of this receptor (Hirasawa et al., 2005; Christiansen et al., 2015), there has been strong interest in better understanding the biologic functions of FFA4 and in its potential as a therapeutic target (Liu et al., 2015; Li et al., 2016). Because reported effects of FFA4 activation include regulation of glucose homeostasis (Oh et al., 2014; Azevedo et al., 2016), regulation of release of incretins and satiety-regulating hormones (Hirasawa et al., 2005), modulation of insulin sensitivity (Oh et al., 2010, 2014; Azevedo et al., 2016), and potential effects on weight gain (Ichimura et al., 2012; Mo et al., 2013; Azevedo et al., 2016), a large focus of studies on FFA4 has centered on the possibility that agonists of this receptor might be useful antidiabetic agents (Cornall et al., 2014; Zhang and Leung, 2014; Moran et al., 2016). However, it is clear that the expression pattern of FFA4, which includes high levels in the lung, in macrophage populations, and in various cancer cell types (Houthuijzen, 2016; Milligan et al., 2017), indicates a broader set of roles and potential for therapeutic interventions targeting this receptor (Milligan et al., 2017). This has resulted in screening for, and the identification of, a number of series of FFA4 agonist ligands (Milligan et al., 2015, 2017), although chemical diversity within these series has, until recently (e.g., Sparks et al., 2014; Azevedo et al., 2016), remained rather limited and based on carboxylate-containing ligands that resemble synthetic fatty acids. FFA4 has been shown to be a potential regulator of adipocyte development and differentiation (Gotoh et al., 2007), potentially via increasing expression of the adipogenic transcription factor peroxisome proliferator activator receptor *γ* (PPAR*γ*) (Hasan et al., 2015; Song et al., 2016). Moreover, adipocyte differentiation from bone marrow mesenchymal stem cells has also been suggested to be promoted by FFA4 (Gao et al., 2015).

A major challenge in studying all aspects of both in vivo and ex vivo regulation of cell and tissue function by FFA4 is that the endogenous fatty acid ligands that are able to activate the receptor are present ubiquitously. Furthermore, being bound extensively by plasma proteins, the concentrations and disposition of “free” fatty acids are often challenging to define (Ulven and Christiansen, 2015), as is the extent of receptor activation in situ that can be attributed to such systemic, circulating fatty acids. As such, alongside various “knockdown” and “knockout” strategies (Alvarez-Curto et al., 2016), well characterized and selective blockers of FFA4 function would be highly valuable tool compounds to further unravel biologic roles of FFA4. To date, a single FFA4 “antagonist” has been described. Compound 39 (Sparks et al., 2014) [now designated AH-7614 (4-methyl-*N*-9*H*-xanthen-9-yl-benzenesulfonamide) by commercial vendors] was shown to block effects of both a fatty acid and a synthetic FFA4 agonist in FFA4-transfected U2OS human osteosarcoma cells, and to antagonize both FFA4-mediated insulin secretion from mouse insulinoma MIN6 cells and the secretion of GLP-1 from the human intestinal cell line NCI-H716 (Sparks et al., 2014).

Herein, we examine the antagonistic properties of AH-7614, and a small number of chemical derivatives, at FFA4. This demonstrates that AH-7614 is able to inhibit FFA4-mediated signals induced by a range of distinct fatty acids and synthetic FFA4 agonist ligands across all endpoints tested. Analysis of the mechanism of antagonism indicates that these compounds are negative allosteric modulators (NAMs) of FFA4 function, which display “probe dependence” in the nature of their inhibitory properties, depending on the specific agonists they are inhibiting. This work also identifies a close chemical derivative of AH-7614 that lacks antagonistic activity at FFA4, which, given the current lack of knowledge about potential off-target effects of AH-7614, will be a useful tool in defining FFA4-specific biologic effects. We have demonstrated the utility of this approach in assessing the role of FFA4 in adipogenesis of murine C3H10T1/2 mesenchymal stem cells.

## Materials and Methods

### 

#### Materials and Compounds.

Unless otherwise stated, all biochemicals and reagents were from Sigma-Aldrich (St. Louis, MO). Tissue culture reagents and buffers were from Life Technologies Inc (Paisley, UK). TUG-891 [4-[(4-fluoro-4'-methyl[1,1'-biphenyl]-2-yl)methoxy]-benzenepropanoic acid] was synthesized as described previously (Shimpukade et al., 2012). Compound 34 (TUG-1197) (2-(3-(pyridin-2-yloxy)phenyl)-2,3-dihydrobenzo[d]isothiazole 1,1-dioxide) was synthesized as described by Azevedo et al. (2016). GSK137647A [4-methoxy-*N*-(2,4,6-trimethylphenyl)-benzenesulfonamide] was synthesized according to Sparks et al. (2014). TUG-770 (4-[2-[2-(cyanomethyl)phenyl]ethynyl]-2-fluorobenzenepropanoic acid) was synthesized as described previously (Christiansen et al., 2013). Cpd A (3-[2-chloro-5-(trifluoromethoxy)phenyl]-3-azaspiro[5.5]undecane-9-acetic acid) was synthesized essentially as described by Chelliah et al. (2014). AH-7614/compound 39 was synthesized as follows: xanthone (100 mg, 0.5 mmol) was dissolved in methanol (10 ml), sodium borohydride (96 mg, 2.6 mmol) was added portionwise over 20 minutes, and the reaction was stirred at room temperature for 24 hours. The reaction was partitioned between ethyl acetate and water. The two phases were separated, and the aqueous phase was extracted twice with ethyl acetate. The organic phases were combined, washed with brine, dried over anhydrous sodium sulfate, filtered, and concentrated. The crude product was used directly in the next step without further purification. *p*-Toluenesulfonamide (95 mg, 0.56 mmol) was added to the crude mixture, followed by acetic acid (5 ml). The reaction was stirred for 2 hours at room temperature. The reaction was partitioned between water and ethyl acetate. The two phases were separated, and the aqueous phase was extracted twice with ethyl acetate. The organic phases were combined, washed with brine, dried over anhydrous sodium sulfate, filtered, and concentrated. The crude product was purified by silica gel flash chromatography (ethyl acetate/petroleum ether, 1:4) to give the desired compound as a white solid (136 mg, 77% over two steps): mp 194.1–195.5°C; ^1^H NMR (400 MHz, CDCl_3_) 7.83–7.75 (m, 2H), 7.33 (d, *J* = 8.0 Hz, 2H), 7.30–7.23 (m, 2H), 7.15 (dd, *J* = 7.8, 1.4 Hz, 2H), 7.08 (dd, *J* = 8.3, 1.0 Hz, 2H), 6.99 (td, *J* = 7.7, 1.2 Hz, 2H), 5.77 (d, *J* = 8.6 Hz, 1H), 4.91 (d, *J* = 8.6 Hz, 1H), 2.47 (s, 3H); ^13^C NMR (100 MHz, CDCl_3_): 151.3, 143.6, 138.6, 129.8, 129.5, 127.2, 123.6, 120.4, 116.7, 49.2, 21.6; electrospray ionization-high-resolution mass spectrometry (ESI-HRMS) *m/z*: calculated C_20_H_17_NaO_3_S [M+Na^+^]: 374.0821; found: 374.0804; high-performance liquid chromatography (HPLC) 99.7%. The data agree with those of Liu et al. (2013). TUG-1387 [4-methyl-*N*-(9*H*-xanthen-9-yl)benzamide] was synthesized as follows: 4-Methylbenzamide (205 mg, 1.5 mmol) and 9*H*-xanthen-9-ol (150 mg, 0.76 mmol) were suspended in acetic acid (1.5 ml) and heated to 90°C. The compounds went into solution after 1 minute, and a few minutes later, a white solid started to precipitate. The reaction was heated overnight and cooled to room temperature, filtered and washed with acetic acid (0.5 ml), then washed with cold ethyl acetate (3 ml). Subsequent drying gave the product as a white solid (180 mg, 75%): mp 229.8–231.6°C; *t*_R_ = 12.88 minutes (HPLC purity: 98.7%); ^1^H NMR [400 MHz, *d*_6_-dimethylsulfoxide (*d*_6_-DMSO)] *δ* 9.36 (d, *J* = 8.6 Hz, 1H), 7.82 (d, *J* = 8.2 Hz, 1H), 7.40 (d, *J* = 7.6 Hz, 1H), 7.37–7.31 (m, 1H), 7.26 (d, *J* = 8.0 Hz, 1H), 7.20–7.11 (m, 1H), 6.55 (d, *J* = 8.6 Hz, 1H), 2.34 (s, 1H); ^13^C NMR (101 MHz, *d*_6_-DMSO) *δ* 165.7, 150.6, 141.3, 131.1, 130.0, 128.9, 128.8, 127.5, 123.4, 121.9, 116.1, 43.0, 20.9; ESI-HRMS calculated for C_21_H_17_NnaO_2_ [M+Na^+^]: 338.1151, found: 338.1163. The data agree with those of Patrick and Dolan (1973). TUG-1506 (4-methyl-*N*-(9*H*-thioxanthen-9-yl)benzenesulfonamide) was synthesized as follows: A 250-ml flask was charged with thioxanthone (2.0 g, 9.4 mmol), tetrahydrofuran (30 ml), and methanol (10 ml). The solution was stirred at room temperature, and NaBH_4_ (1.07 g, 28.3 mmol) was added slowly. The initially yellow reaction mixture slowly turned orange. After 45 minutes, the solution was diluted with water and extracted three times with ethyl acetate. The combined organic phases were washed with brine, dried over sodium sulfate, and concentrated to give the crude alcohol as an orange solid (2.02 g) that was used immediately in the next step: ^1^H NMR (400 MHz, *d*_6_-DMSO) *δ* 7.70 (d, *J* = 7.6 Hz, 2H), 7.50 (dd, *J* = 7.7, 0.9 Hz, 2H), 7.36 (td, *J* = 7.5, 1.2 Hz, 2H), 7.28 (td, *J* = 7.5, 1.0 Hz, 2H), 6.40 (d, *J* = 5.8 Hz, 1H), 5.19 (d, *J* = 5.7 Hz, 1H); ^13^C NMR (101 MHz, *d*_6_-DMSO) *δ* 139.0, 130.8, 126.8, 126.6, 126.4, 125.3, 69.3. The data agree with those of Cozzi and Zoli (2008). 9*H*-Thioxanthen-9-ol (2.02 g, 9.4 mmol) and *p*-toluenesulfonamide (2.22 g, 10.4 mmol) were dissolved in acetic acid (30 ml) and stirred at room temperature overnight. The resulting precipitate was isolated by filtration and washed first with cold acetic acid and then cold petroleum ether to afford the product as a white solid (2.2 g, 64%): mp 158.7–163.9°C; *t*_R_ = 12.94 minutes (HPLC purity: 99.0%); *R_f_* = 0.40 (EtOAc:PE, 1:1); ^1^H NMR (400 MHz, *d*_6_-DMSO) *δ* 8.76 (d, *J* = 8.4 Hz, 1H), 7.65 (d, *J* = 8.3 Hz, 2H), 7.50–7.46 (m, 2H), 7.37–7.28 (m, 4H), 7.28–7.19 (m, 4H), 5.23 (d, *J* = 8.4 Hz, 1H), 2.35 (s, 3H); ^13^C NMR (101 MHz, *d*_6_-DMSO) *δ* 142.4, 138.8, 134.9, 132.2, 129.3, 127.3, 126.8, 126.7, 126.5, 126.3, 56.5, 20.9; ESI-HRMS calculated for C_20_H_17_NNaO_2_S_2_ [M+Na^+^]: 390.0593, found: 390.0585. The data agree with those of Tamura et al. (1977).

#### Cell Culture.

HEK293T cells were maintained in Dulbecco’s modified Eagle’s medium (DMEM) supplemented with 10% (v/v) fetal bovine serum (FBS) at 37°C and 5% (v/v) CO_2_. Flp-In T-REx 293 cell lines, generated to inducibly express various tagged versions of human FFA4 (hFFA4), human FFA1 (hFFA1), or murine FFA4 (mFFA4) following treatment with doxycycline, were maintained in DMEM supplemented with 10% (v/v) FBS, 100 U/ml penicillin, 100 *μ*g/ml streptomycin, and 200*μ*g/ml hygromycin B. Human brain astrocytoma 1321N1 cells stably transfected with hFFA1 were grown in DMEM supplemented with 10% (v/v) FBS, 100 U/ml penicillin, 100 *μ*g/ml streptomycin, and 400 *µ*g/ml G418. To study adipogenesis, murine C3H10T1/2 cells (Tang et al., 2004) were grown in DMEM containing 10% (v/v) FBS, 100 U/ml penicillin, and 100 *μ*g/ml streptomycin in a 37°C incubator with 5% (v/v) CO_2_. Cells in 10-cm dishes were differentiated with IID [100 nM insulin, 500 *µ*M 3-isobutyl-1-methylxanthine (IBMX), and 10 nM dexamethasone] for 5 days in the presence/absence of vehicle [0.1% (v/v) DMSO], 10 *µ*M AH-7614, or 10 *µ*M TUG-1387.

#### Plasmids.

Plasmids encoding either the human (short isoform) or mouse FFA4 receptors with enhanced yellow fluorescent protein (eYFP) or the related fluorescent protein mVenus fused to their C terminus and incorporating an N-terminal FLAG epitope tag in the pcDNA5 FRT/TO expression vector were generated as previously described (Hudson et al., 2013; Butcher et al., 2014; Alvarez-Curto et al., 2016; Prihandoko et al., 2016).

#### *β*-Arrestin-2 Interaction Assay.

*β*-Arrestin-2 recruitment to either human or mouse isoforms of FFA4 was measured using a bioluminescence resonance energy transfer (BRET)–based approach. HEK293T cells were cotransfected with eYFP-tagged forms of each receptor in a 4:1 ratio with a *β*-arrestin-2 *Renilla* luciferase plasmid using polyethylenimine. Cells were then transferred into white 96-well plates at 24 hours post-transfection. At 48 hours post-transfection, cells were washed with Hanks’ balanced salt solution (HBSS) and then incubated in fresh HBSS prior to the assay. Cells were preincubated for 15 minutes with HBSS supplemented with vehicle [1% (v/v) DMSO], AH-7614, TUG-1506, or TUG-1387. Cells were incubated with 2.5 *µ*M *Renilla* luciferase substrate coelenterazine h (Nanolight Tech, Pinetop, CA) at 37°C for 10 minutes and then stimulated with various FFA4 agonists for a further 5 minutes at 37°C. BRET resulting from receptor–*β*-arrestin-2 interaction was then determined by measuring the ratio of luminescence at 535 and 475 nm using a Pherastar FS fitted with the BRET1 optic module (BMG Labtech, Aylesbury, UK).

#### Ca^2+^ Mobilization.

Calcium assays were carried out on either Flp-In T-REx 293 cell lines, generated to inducibly express hFFA4 upon treatment with doxycycline, or 1321N1 cells stably expressing the hFFA1 receptor. One day prior to the experiment, cells were seeded at 50,000 cells/well (Flp-In REx 293) or 25,000 cells/well (1321N1) in poly-d-lysine–coated, black, clear-bottom, 96-well microplates. Cells were allowed to adhere for 3–4 hours before the addition of 100 ng/ml doxycycline to induce receptor expression in the case of Flp-In T-REx 293 cell lines. The following day, cells were incubated in culture medium containing the calcium-sensitive dye Fura2-AM (3 *μ*M) for 45 minutes. Cells were then washed three times and then allowed to equilibrate for 15 minutes in HBSS prior to conducting the assay. Fura2 fluorescent emission was measured at 510 nm following excitation at both 340 and 380 nm during the course of the experiment using a Flexstation plate reader (Molecular Devices, Brambleside, UK). Calcium responses were then measured as the difference between 340/380 ratios before and after the addition of the relevant compounds. For antagonism, the cells were preincubated for 15 minutes prior to agonist addition with HBSS supplemented with vehicle [1% (v/v) DMSO], AH-7614, or TUG-1387 (10 *μ*M) prior to the addition of 50 *µ*M *α* linolenic acid (aLA), 500 nM TUG-891, or 13 nM TUG-770.

#### High-Content Imaging Quantitative Internalization Assay.

hFFA4-mVenus Flp-In T-REx 293 cells were plated 75,000 cells/well in black with clear-bottom 96-well plates. Cells were allowed to adhere for 3–6 hours before the addition of doxycycline (100 ng/ml) to induce receptor expression. After an overnight incubation, culture medium was replaced with serum-free DMEM containing the ligand to be assessed and incubated at 37°C for 30 minutes before fixation with 4% paraformaldehyde. After washing with phosphate-buffered saline, cell nuclei were stained for 30 minutes with Hoechst33342.

Plates were subsequently imaged using a Cellomics ArrayScan II high-content plate imager (Thermo Fisher Scientific, Paisley, UK). Images were processed to identify internalized mVenus, which was then normalized to cell number based on nuclei identified by Hoechst33342 staining, to obtain a quantitative measure of hFFA4-mVenus internalization.

#### HTRF-Based Inositol Monophosphate Assay.

Inositol monophosphate assays (Cisbio Bioassays, Codolet, France) were performed according to the manufacturer’s instructions. In brief, a suspension of 7500 cells/well was incubated with the stated concentrations of agonist for 1 hour in the presence of 10 mM lithium chloride (LiCl). Inositol monophosphate accumulation was subsequently measured using a Pherastar FS plate reader.

#### RNA Isolation and Reverse-Transcription Quantitative Polymerase Chain Reaction (RT-qPCR).

Total RNA was isolated from C3H10T1/2 cells using an RNEasy mini kit (Qiagen, Manchester, UK). Following RNA isolation, reverse-transcription polymerase chain reaction was performed using Superscript III (Life Technologies), and the resultant cDNA was used as a template for quantitative polymerase chain reaction analysis using an ABI Prism 7300 sequence detector (Applied Biosystems, Paisley, UK). Cycling conditions were as follows: 50°C for 2 minutes, 95°C for 10 minutes, followed by 40 cycles of 95°C for 15 seconds and 60°C for 1 minute. FFA4, PPAR*γ*, and Runx2 expressions were then defined relative to cyclophilin using the 2^−ΔΔCt^ method.

#### Western Blotting.

Analysis of receptor phosphorylation was performed on mFFA4-eYFP cells that were pretreated with either 10 *µ*M AH-7614 or TUG-1387 for 30 minutes, after which time they were treated with 10 *µ*M TUG-891 for 5 minutes. Cell lysates were prepared and size fractionated on 4–12% SDS-PAGE gels and transferred to nitrocellulose membranes. Nitrocellulose membranes containing resolved receptor proteins were incubated in Tris-buffered saline LI-COR blocking buffer (LI-COR Biosciences, Cambridge, UK) and incubated subsequently with a phospho-specific antiserum produced in house that is able to recognize phosphorylated Thr^347^ and Ser^350^ residues of the receptor (Prihandoko et al., 2016). Membranes were air-dried and scanned using an LI-COR Odyssey CLx Imager.

#### Oil Red O Staining.

Untreated or differentiated C3H10T1/2 cells were fixed in 10% (v/v) formalin for 60 minutes at room temperature. The cells were washed in 60% (v/v) isopropanol and then incubated for 10 minutes with isopropanol supplemented with 0.6% (w/v) Oil Red O. The Oil Red O solution (Sigma-Aldrich) was then removed, and cells were washed ×4 with dH_2_O and then stored in phosphate-buffered saline at 4°C until visualization using an Invitrogen EVOS FL Auto Imaging System (Life Technologies). After removal of all liquid, the Oil Red O stain was dissolved in isopropanol and the absorbance measured at 405 nm using a Pherastar FS reader.

#### Statistical Analysis.

Data are presented as the mean ± S.E.M. of a minimum of three independent experiments performed in triplicate, unless otherwise stated in the respective figure legends. Data analysis was carried out using GraphPad Prism software (package v5.02; GraphPad Software, La Jolla, CA), with concentration-response data fitted to three-parameter sigmoidal curves. For allosteric modulation experiments, data sets were globally fit to an operational model of allosterism described previously (Hudson et al., 2014). To fit these data, the *τ* value for all allosteric modulators was constrained to reflect the lack of detectable agonism seen within these compounds, which allowed for curve fits yielding estimates of log*α*, log*β*, logK_A_, and logK_B_. Statistical analyses were carried out using one-way analysis of variance followed by Tukey’s post-hoc test.

## Results

**Fig. 1.**
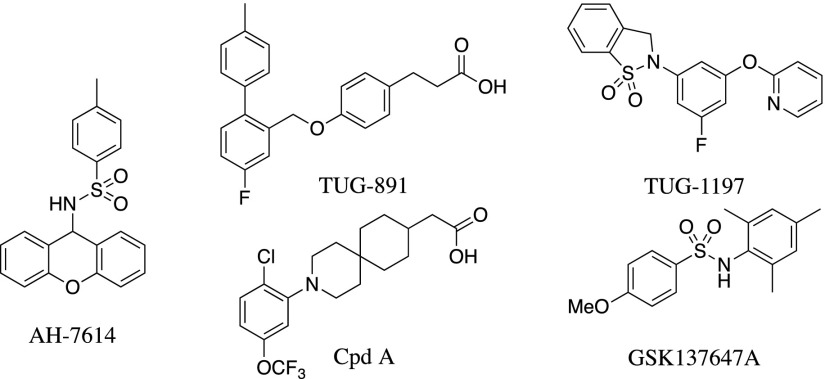
The chemical structures of the FFA4 antagonist AH-7614 and a range of FFA4 agonists (TUG-891, Cpd A, GSK137647A, and TUG-1197).

**Fig. 2.**
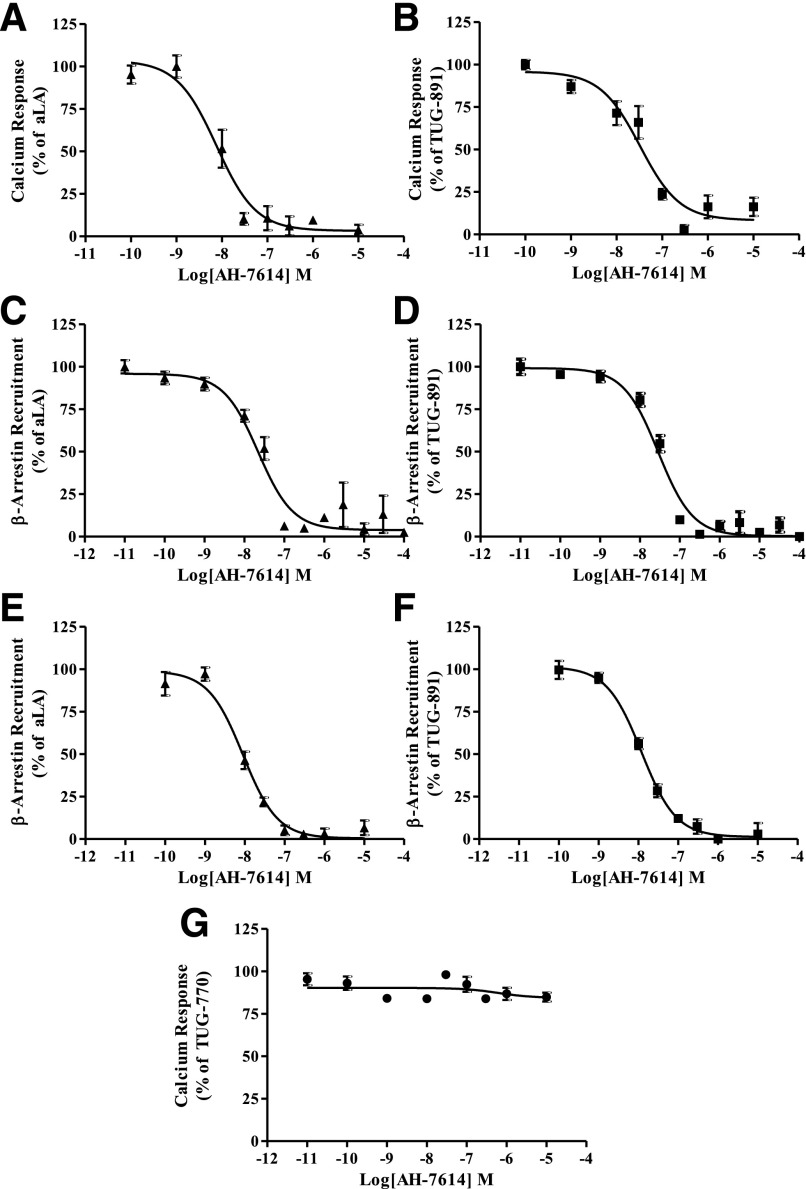
AH-7614 is a potent and selective inhibitor of both human and murine FFA4 function. Flp-In T-REx 293 cells induced to express hFFA4-eYFP were pretreated with either vehicle [1% (v/v) DMSO] or increasing concentrations of AH-7614 for 15 minutes, after which time they were treated with either 50 *µ*M aLA (A) or 500 nM TUG-891 (B). Both aLA and TUG-891 induced calcium release that was potently inhibited in the presence of AH-7614 (aLA, pIC_50_ = 7.51 ± 0.08, *n* = 3; TUG-891, pIC_50_ = 8.13 ± 0.08, *n* = 3). HEK293T cells transiently expressing hFFA4-eYFP and *β*-arrestin-2-*Renilla* luciferase were preincubated with either vehicle [1% (v/v) DMSO] or increasing concentrations of AH-7614. The cells were then treated with either 50 *µ*M aLA (C) or 500 nM TUG-891 (D), and *β*-arrestin-2 recruitment was subsequently determined using a BRET-based assay. Both aLA and TUG-891 promoted *β*-arrestin-2 recruitment that was potently inhibited (aLA, pIC_50_ = 7.66 ± 0.05, *n* = 5; TUG-891, pIC_50_ = 7.55 ± 0.07, *n* = 5) in the presence of increasing concentrations of AH-7614. mFFA4-dependent *β*-arrestin-2 recruitment in the presence of either 50 *µ*M aLA (E) or 500 nM TUG-891 (F) was also potently inhibited (aLA, pIC_50_ = 8.05 ± 0.08 *n* = 3; TUG-891, pIC_50_ = 7.93 ± 0.06, *n* = 3) by AH-7614. However, AH-7614 had no effect on TUG-770–induced calcium release in 1321N1 cells stably transfected with hFFA1 receptor (G), indicating that AH-7614 is not an antagonist at this receptor. Data represent experiments performed in triplicate three times or more.

We synthesized and assessed the functional activity of AH-7614 (Fig. 1), a xanthene derivative of a diarylsulfonamide-based FFA4 agonist. This compound was originally described as an antagonist of FFA4, able to block effects of both the polyunsaturated *ω*-6 fatty acid linoleic acid and the synthetic FFA4 agonist GSK137647A (Fig. 1) (Sparks et al., 2014), and has since been used in a number of functional studies (Quesada-López et al., 2016; Houthuijzen et al., 2017; Villegas-Comonfort et al., 2017) but without analysis of its mode of action. Indeed, AH-7614 inhibited, in a potent and concentration-dependent manner (pIC_50_ vs. EC_80_ concentrations of the agonists), the ability of either the *ω*-3 fatty acid aLA (pIC_50_ 7.51 ± 0.08, *n* = 3) or a synthetic orthobiphenyl-based agonist of FFA4, TUG-891 (Fig. 1) (pIC_50_ 8.13 ± 0.08, *n* = 3) (Shimpukade et al., 2012; Hudson et al., 2013; Butcher et al., 2014), to promote Ca^2+^ mobilization in Flp-In T-REx 293 cells induced to express hFFA4-eYFP (Fig. 2, A and B). Similar results were obtained when measuring the ability of AH-7614 to inhibit agonist-induced interactions of hFFA4 with *β*-arrestin-2 following coexpression of these two proteins in HEK293T cells (Fig. 2, C and D). Importantly for subsequent studies, AH-7614 also functionally antagonized effects of both aLA and TUG-891 at the corresponding mouse ortholog of the receptor (mFFA4) (Fig. 2, E and F). AH-7614 showed selectivity for FFA4, as it was unable to antagonize activation of the other long-chain fatty acid receptor, FFA1 (Milligan et al., 2015, 2017), in studies in which the synthetic alkyne-based FFA1 agonist TUG-770 (Christiansen et al., 2013) promoted elevation of intracellular Ca^2+^ in 1321N1 cells stably transfected to express a human FFA1 construct (Fig. 2G).

**Fig. 3.**
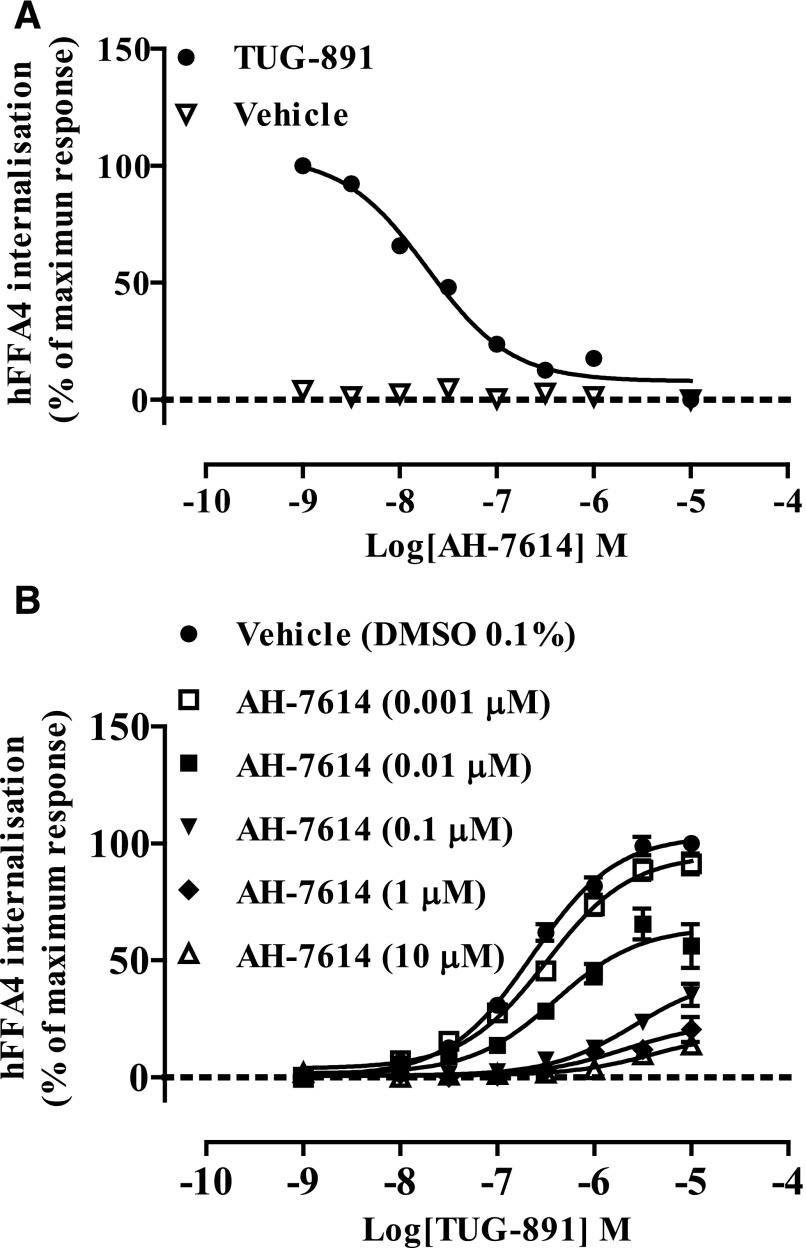
AH-7614 blocks agonist-induced receptor internalization of the human FFA4 receptor. Flp-In T-REx 293 cells induced to express hFFA4-mVenus were pretreated with 10 *µ*M AH-7614 for 15 minutes, after which time cells were further treated with either vehicle [0.1% (v/v) DMSO] or TUG-891 (3 *µ*M) for 30 minutes (A) and subsequently fixed. AH-7614 prevented TUG-891 induced receptor internalization (pIC_50_ = 7.70 ± 0.10). Cells were pretreated with varying concentrations of AH-7614 and exposed to increasing concentrations of TUG-891 (B) for 30 minutes, after which they were fixed. Cells were imaged using a Cellomics ArrayScan II, and the extent of internalization of the receptor construct was quantified.

In similar EC_80_ inhibition experiments, AH-7614 was also effective in blocking TUG-891–mediated internalization of FFA4 from the cell surface (Fig. 3A) (pIC_50_ = 7.70 ± 0.10, *n* = 3). We therefore used this internalization assay, with an endpoint 30 minutes after addition of agonist to facilitate achievement of ligand equilibrium, to probe the mechanism of AH-7614–mediated blockade of FFA4. Receptor internalization experiments were conducted in which the ability of defined concentrations of preadded AH-7614 to block internalization in response to various concentrations of TUG-891 was assessed (Fig. 3B). In these experiments, it was clear that the primary inhibitory effect of AH-7614 was to produce a decrease in the maximal response to TUG-891. Although this is consistent with outcomes reported by Sparks et al. (2014) using intracellular Ca^2+^ assays, the longer time course of the internalization studies largely eliminates potential issues of ligand hemiequilibrium that can greatly influence outcomes and mechanistic interpretation in assays, such as those based on Ca^2+^ mobilization, that use much shorter endpoints (Charlton and Vauquelin, 2010). These findings indicated that AH-7614 is not a competitive antagonist of FFA4. Interestingly, even at the highest concentration of AH-7614 tested, a residual response to TUG-891 remained. This response to TUG-891 was similar in the presence of 1 and 10 *μ*M AH-7614, and as such, the effect of AH-7614 appeared to saturate. Such an outcome is consistent with AH-7614 functioning as an NAM of FFA4.

**Fig. 4.**
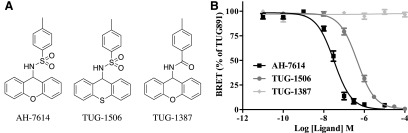
TUG-1387 and TUG-1506 are chemical derivatives of AH-7614 with varying activity at FFA4. (A) Chemical structures of AH-7614, TUG-1387, and TUG-1506. HEK293T cells transiently coexpressing hFFA4-eYFP and *β*-arrestin-2–*Renilla* luciferase were preincubated with vehicle (0.1% DMSO) or increasing concentrations of AH-7614, TUG-1387, or TUG-1506. *β*-Arrestin-2 recruitment to the receptor was then determined in the presence 500 nM TUG-891 (B). All data represent experiments carried out in triplicate at least three times.

As it is common for small chemical changes in allosteric GPCR ligands to significantly alter the details of their allosteric behavior (Wood et al., 2011; Hudson et al., 2014), we next synthesized derivatives of AH-7614 and assessed their activity at FFA4. Two key derivatives were considered. In the first, the sulfonamide of AH-7614 was replaced with an amide (TUG-1387), whereas in the second, the xanthine was replaced with a thioxanthene (TUG-1506) (Fig. 4A). These analogs were tested for their ability to antagonize EC_80_ concentrations of TUG-891 (Fig. 4B) in a *β*-arrestin-2 recruitment assay. The thioxanthene-containing TUG-1506 retained the ability to inhibit TUG-891 (pIC_50_ = 6.38 ± 0.09, *n* = 3) but with lower potency than the xanthene containing AH-7614. In contrast, the amide-containing TUG-1387 lacked all ability to inhibit TUG-891–mediated activation of FFA4.

**Fig. 5.**
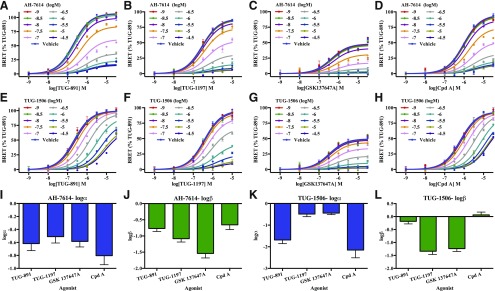
AH-7614 and TUG-1506 are probe-dependent negative allosteric modulators of FFA4. The nature of AH-7614 (A–D) and TUG-1506 (E–H) allosteric modulation of FFA4 function was probed by their ability to limit recruitment of *β*-arrestin-2 to hFFA4-eYFP induced by varying concentrations of four chemically distinct FFA4 agonists [TUG-891 (A and E), TUG-1197 (B and F), GSK137647A (C and G), and Merck compound A (Cpd A) (D and H)] using HEK293T cells transiently coexpressing hFFA4-eYFP and *β*-arrestin-2–*Renilla* luciferase. Data are fitted to an operational model of allosterism (Hudson et al., 2014). Estimated parameters for log*α* and log*β* are shown for both AH-7614 (I and J) and TUG-1506 (K and L) when assessed at each of the four agonists. Data shown are from representative experiments (N numbers for each agonist-NAM combination are reported in Table 1) fit to an operational model of allosterism (Hudson et al., 2014).

As AH-7614, and likely TUG-1506, appeared to be NAMs of FFA4 function, we characterized and quantified the nature and extent of their NAM properties against synthetic FFA4 agonists from several distinct chemotypes. A common feature of GPCR allosteric modulators is that they may show “probe dependence,” producing different allosteric effects depending on the orthosteric agonist being modulated (May et al., 2007). Therefore, we conducted experiments in which AH-7614 (Fig. 5, A–D) and TUG-1506 (Fig. 5, E–H) were used to antagonize *β*-arrestin-2 recruitment to FFA4 induced by synthetic agonists from each of four distinct chemotypes: TUG-891 (Shimpukade et al., 2012), TUG-1197 (Azevedo et al., 2016), GSK137647A (Sparks et al., 2014), and Cpd A (Oh et al., 2014) (Fig. 1). Data from these experiments were then fit to an operational model of allosterism to quantify the allosteric effect each NAM had on affinity of each orthosteric agonist (*α*), the allosteric effect on efficacy of each orthosteric agonist (*β*), the affinity of the orthosteric agonists (K_A_), and the affinity of the modulator (K_B_) (Table 1). AH-7614 reduced the maximal response to each agonist tested, and produced a modest reduction in agonist potency (Fig. 5, A–D). This corresponded to log*α* (Fig. 5I; Table 1) and log*β* (Fig. 5J; Table 1) values that were both less than zero for each of the four agonists. However, although the log*α* values were broadly similar for each agonist, the log*β* for GSK137647A was significantly lower (*P* < 0.01) than the value obtained for either TUG-891 or Cpd A, suggesting a subtle level of probe dependence in AH-7614 modulation of FFA4 activity. This can be noted in the concentration-response curves by the observation that, although high concentrations of AH-7614 eliminated measureable response to GSK137647A (Fig. 5C), a residual response was apparent for all other agonists, even at 30 *μ*M AH-7614 (Fig. 5, A, B, and D). Importantly, the agonist responses were similar in the presence of between 3 and 30 *μ*M AH-7614, indicating that the inhibitory properties of AH-7614 were saturable, a hallmark of allosteric modulators.

**TABLE 1:** Operational model curve-fit parameters for AH-7614 and TUG-1506

Agonist	log*α*	log*β*	pK_A_	pK_B_	*N*
AH-7614					
TUG-891	−0.80 ± 0.11	−0.77 ± 0.09	6.77 ± 0.14	7.98 ± 0.24	4
TUG-1197	−0.52 ± 0.09	−1.08 ± 0.10	6.44 ± 0.05	7.82 ± 0.26	5
GSK137647A	−0.58 ± 0.09	−1.54 ± 0.14	6.32 ± 0.04	8.14 ± 0.21	3
Cpd A	−0.80 ± 0.14	−0.66 ± 0.15	6.31 ± 0.22	8.17 ± 0.26	3
TUG-1506					
TUG-891	−1.68 ± 0.17	−0.18 ± 0.10	6.79 ± 0.11	7.46 ± 0.06	4
TUG-1197	−0.48 ± 0.12	−1.34 ± 0.13	6.48 ± 0.08	6.90 ± 0.04	3
GSK137647A	−0.44 ± 0.07	−1.24 ± 0.11	6.44 ± 0.03	7.04 ± 0.10	3
Cpd A	−2.15 ± 0.36	0.06 ± 0.11	6.46 ± 0.18	7.28 ± 0.02	3

**Fig. 6.**
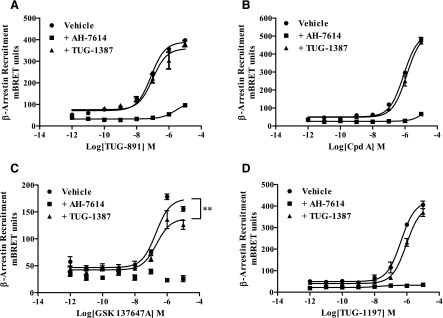
TUG-1387 does not antagonize the effects of distinct FFA4 agonist chemotypes. HEK293T cells transiently expressing mFFA4-eYFP and *β*-arrestin-2–*Renilla* luciferase constructs were pretreated for 30 minutes with vehicle, 10 *µ*M AH-7614, or 10 *µ*M TUG-1387. They were then treated with TUG-891 (A), Cpd A (B), GSK137647A (C), or TUG-1197 (D) to assess receptor-mediated arrestin recruitment. AH-7614 was able to block *β*-arrestin-2 recruitment in all cases, whereas TUG-1387 did not have any effect except to produce a small but significant (**P < 0.001) reduction of the efficacy of GSK137647A (C). Data represent experiments performed in triplicate three/four times. mBRET, units are equal to the 535/475 nm emission ratio multiplied by 1000.

In examining the allosterism between TUG-1506 and the various FFA4 agonists (Fig. 5, E–H), much more striking probe dependence was apparent. Most notably, whereas TUG-1506 primarily shifted the potency of TUG-891 (Fig. 5E) and Cpd A (Fig. 5H) with little effect on maximal response, the opposite was true for TUG-1197 and GSK137647A, where TUG-1506 depressed the maximal response without affecting agonist potency. This was reflected in the operational model curve fit parameters for these experiments, where a large negative log*α* was observed for TUG-891 and Cpd A (Fig. 5K; Table 1) but a large negative log*β* was observed for TUG-1197 and GSK137647A (Fig. 5L; Table 1). This is a clear indication of the probe dependence in the NAM properties of TUG-1506. Interestingly, this may be related to the chemical nature of the agonists, with TUG-891 and Cpd A both being carboxylate-based FFA4 agonists, whereas TUG-1197 and GSK137647A are each sulfonamide-based agonists. Importantly, once again, the inhibitory effects of TUG-1506 were saturable in all cases, entirely consistent with defining this compound as an NAM at FFA4. Similar experiments were also conducted with TUG-1387 against each FFA4 agonist, although in this case, given that TUG-1387 had already been shown to lack activity at FFA4, only a single high concentration of TUG-1387 was used (Fig. 6, A–D). TUG-1387 did not produce an NAM effect on TUG-891, TUG-1197, or Cpd A, and only produced a very modest effect on GSK137647A. Overall, these results are consistent with either TUG-1387 having no affinity for FFA4 or it being a silent allosteric modulator that, even if it does bind the receptor, does not affect orthosteric agonist function.

**Fig. 7.**
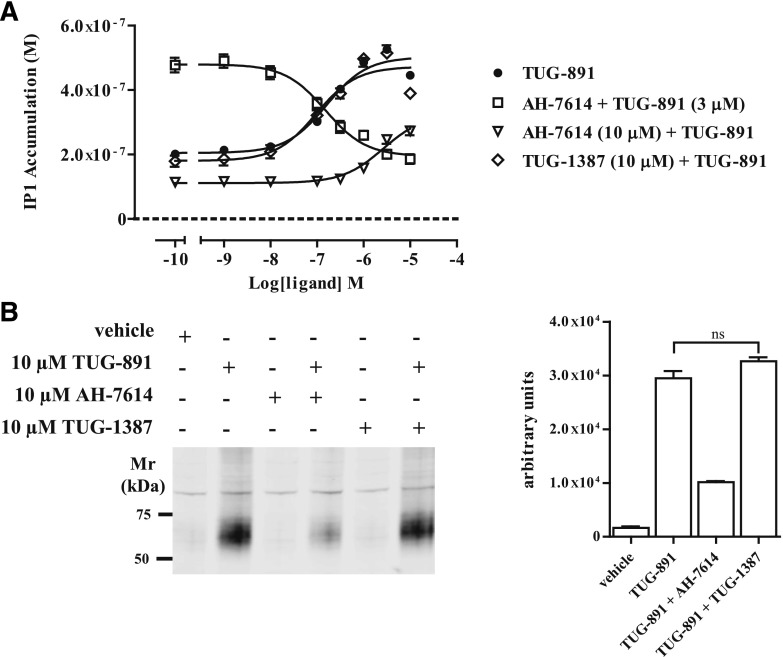
AH-7614, but not TUG-1387, blocks agonist-induced elevation of intracellular inositol monophosphates and phosphorylation of FFA4. (A) FFA4-mediated inositol monophosphate (IP1) production was measured in cells expressing hFFA4-mVenus. TUG-891 (closed circles) promoted accumulation of IP1. Pretreatment with either 10 *µ*M AH-7614 (inverted triangles) or increasing concentrations of this molecule (open squares) blocked IP1 accumulation. By contrast, pretreatment with TUG-1387 was without effect (open diamonds). (B) mFFA4-eYFP cells were pretreated with vehicle or various concentrations of AH-7614 or TUG-1387 for 30 minutes followed by treatment with 10 *µ*M TUG-891 for 5 minutes. Cell lysates were prepared and resolved by SDS-PAGE and immunoblotted with an mFFA4-specific phospho-antiserum that recognizes phosphorylation of residues Thr^347^ and Ser^350^ (Prihandoko et al., 2016). Quantification of a series of such immunoblots is shown in the right-hand panel. ns, *P* > 0.05.

To further assess the potential for using AH-7614 in combination with TUG-1387 to define biologic functions of FFA4, we aimed to confirm the broader ability of AH-7614, but not TUG-1387, to block additional FFA4 signaling pathways. We explored both FFA4-mediated accumulation of inositol monophosphates (Fig. 7A) and agonist-induced phosphorylation of Thr^347^ and Ser^350^ residues within the C terminus of FFA4 detected with previously described phospho-FFA4 antisera (Butcher et al., 2014; Prihandoko et al., 2016) (Fig. 7B). In each case, the agonist response was functionally inhibited by AH-7614, but was not affected by TUG-1387. Importantly for studies on more physiologically relevant systems, we also found that TUG-1387 lacked activity at the related fatty acid receptor FFA1 in experiments measuring calcium release in 1321N1-hFFA1 cells (not shown).

**Fig. 8.**
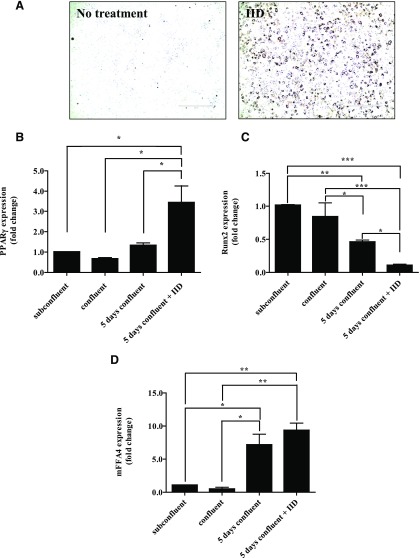
mRNA levels of FFA4 and PPAR*γ* are upregulated in C3H10T1/2 cells by differentiation toward the adipocyte phenotype. (A) C3H10T1/2 cells were differentiated toward an adipocyte phenotype by treatment with IID medium. Confirmation of effective differentiation after 5 days of treatment was obtained by staining for triglyceride droplets within the cells using Oil Red O. RT-qPCR was performed to assess changes in expression levels of PPAR*γ* (B), Runx2 (C), and mFFA4 (D) under conditions of subconfluence, confluence, 5 days postconfluence, and 5 days postconfluence in the presence of IID. Data are presented as the group mean ± S.E.M. of three independent experiments (*different at *P* < 0.05, **P < 0.01, and ***P < 0.001). Scale bar in (A) = 400 *μ*m.

**Fig. 9.**
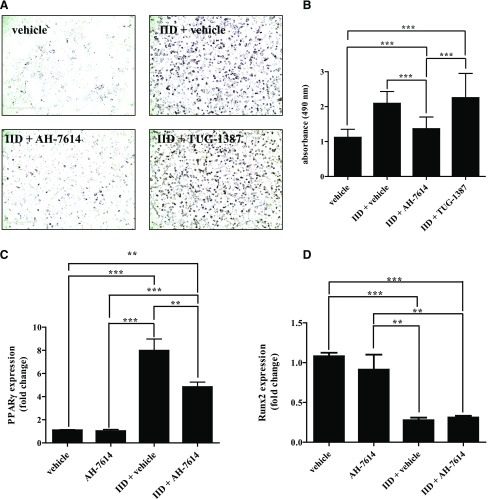
Adipocyte differentiation of C3H10T1/2 cells requires FFA4 signaling. (A) C3H10T1/2 cells were differentiated (IID) in the presence of vehicle (0.1% DMSO), 10 *μ*M AH-7614, or 10 *μ*M TUG-1387 compared with a nondifferentiated control and then stained with Oil Red O. (B) Oil Red O staining was subsequently dissolved in isopropanol and then quantified by measuring absorbance at 405 nm (*n* = 10, data presented as mean ± S.E.M.). RT-qPCR analysis of PPAR*γ* (*n* = 5) (C) and Runx2 (*n* = 4) (D) levels was then performed on differentiated (IID) and nondifferentiated (vehicle) C3H10T1/2 cells in the presence or absence of 10 *µ*M AH-7614. Data are presented as the mean ± S.E. (**P < 0.01, and ***P < 0.001). Scale bar in (A) = 400 *μ*m.

Recently, it has been suggested that FFA4 is able to act as a bipotential regulator of osteogenic and adipogenic differentiation of bone marrow–derived mesenchymal stem cells (Gao et al., 2015). Furthermore, FFA4 has been proposed to be a key player in the activation of brown adipose tissue (Quesada-Lopez et al., 2016), further reinforcing a role for FFA4 in adipose tissue physiology. To further assess the role of FFA4 in adipogenic differentiation, and to demonstrate the utility of the FFA4 NAM (AH-7614) when used in combination with TUG-1387 to interrogate FFA4 biology, we used the murine mesenchymal stem cell line C3H10T1/2 (Tang et al., 2004; Hashimoto et al., 2016; Lee et al., 2016). Here, differentiation toward an adipocyte phenotype was produced by maintaining the cells in the presence of an induction mixture (IID) consisting of 100 nM insulin, 500 *µ*M IBMX, and 10 nM dexamethasone for 5 days. Oil Red O staining effectively visualized the development of triglyceride deposits associated with adipogenesis (Fig. 8A). In concert, exposure to the IID-containing medium produced clear upregulation of mRNA for the adipogenic development marker, PPAR*γ* (Fig. 8B). This effect was not produced simply by cell confluence on the tissue culture plate (Fig. 8B). In parallel, levels of Runt-related transcription factor 2 (Runx2) mRNA, a key transcription factor associated with osteoblast differentiation (Xu et al., 2015; Yuan et al., 2016), were greatly reduced (Fig. 8C). Over this time, frame levels of mFFA4 mRNA were also markedly increased by cell maintenance in the IID medium; interestingly, however, an equivalent upregulation of mFFA4 mRNA was also produced simply by maintaining C3H10T1/2 cells at confluence for a 5-day period (Fig. 8D). The ability of C3H10T1/2 cells to differentiate toward the adipocyte phenotype was clearly dependent upon signaling from FFA4, because treatment of cells with AH-7614 along with the IID medium greatly reduced visual observation of triglyceride stores as detected by Oil Red O staining and its quantification (Fig. 9, A and B). Treatment with AH-7614 also limited IID-induced PPAR*γ* mRNA induction (Fig. 9C), without affecting the associated downregulation of Runx2 expression (Fig. 9D). In contrast, treatment of C3H10T1/2 cells with TUG-1387 during the induction period with the IID-containing medium had no significant effect on the development of Oil Red O staining (Fig. 9, A and B). Taken together, these data indicate that TUG-1387, although structurally related, lacks activity at both FFA4 and the related FFA1, and therefore indicate this compound will be a useful chemical control to confirm that biologic effects produced by AH-7614 and related compounds, such as TUG-1506, are in fact FFA4-mediated.

## Discussion

The availability of pharmacological tool compounds is central to efforts to define functions of many cellular proteins, including GPCRs, and to assess such proteins as potential therapeutic targets. However, for poorly and recently characterized receptors, such tool compounds are often lacking; described largely in the chemical/patent literature and, therefore, often of limited availability; and/or are poorly characterized in terms of selectivity and potential off-target effects. Within the group of GPCRs that respond to nonesterified, or “free,” fatty acids, the most broad-reaching pharmacopoeia to date targets the long-chain fatty acid receptor FFA1 (Milligan et al., 2017). This reflects that FFA1 has been targeted therapeutically and in human clinical trials to assess whether activation of this receptor might provide an approach to treat type II diabetes (Watterson et al., 2014; Li et al., 2016; Mohammad, 2016; Ghislain and Poitout, 2017). Although the second GPCR that is activated by longer-chain free fatty acids, FFA4, is also frequently suggested as a potential therapeutic target for diabetes and other metabolic disorders (Li et al., 2016; Moniri, 2016; Hansen and Ulven, 2017), this lags behind studies on FFA1, and no synthetic ligands with activity at this receptor have yet entered clinical studies (Milligan et al., 2017). As a result, even tool compounds that are useful for assessing roles of this receptor are less widely available or understood (Hansen and Ulven, 2017). Moreover, as clear consensus is based on the therapeutic potential of agonism of FFA4 (Li et al., 2016; Moniri, 2016), little effort has been expended to identify, report, or characterize antagonists of this receptor, despite antagonist tool compounds being a traditional touchstone for detailed pharmacological analysis.

The only inhibitor of FFA4 function reported to date is AH-7614 (Sparks et al., 2014). Although Sparks et al. (2014) suggested that this compound would likely act as a competitive antagonist of FFA4, the limited data presented in their report did not actually suggest such a mode of action, which appeared to be noncompetitive, at least in assays measuring regulation of intracellular Ca^2+^ levels. The studies reported here expand on this, defining AH-7614 as an NAM of FFA4 function. This conclusion is supported by several hallmarks of allosterism (May et al., 2007), including that the ability of AH-7614 to inhibit FFA4 function is saturable, and that AH-7614 displays some level of probe dependence. More striking, however, in this regard is the chemical derivative of AH-7614, TUG-1506, described for the first time in these studies, which shows clear probe dependence depending on the chemical nature of the FFA4 agonist it is modulating. Specifically, this compound is an NAM primarily of ligand affinity for the two carboxylate-containing agonists tested, but primarily an NAM of efficacy for the noncarboxylate, sulphonamide-based agonists. Such changes in allosteric modulation are commonly observed with small chemical changes in allosteric ligands (Wood et al., 2011; Hudson et al., 2014), and indeed, the observed pattern of inhibition by both AH-7614 and TUG-1506 can only be explained by an allosteric mode of action.

Whereas Sparks et al. (2014) more broadly assessed the selectivity of the FFA4 agonist GSK137647A described in their study, they did not extend this to AH-7614. Therefore, having identified TUG-1387 in the current work as a close structural analog of AH-7614 that lacks activity at FFA4, we used each of these ligands to define specific roles for FFA4. Indeed, the FFA4 NAM AH-7614 prevents differentiation of a mouse mesenchymal stem cell line toward an adipocyte phenotype, and does so in a manner that is consistent with blockade of endogenous signaling via FFA4.

We describe AH-7614 and TUG-1506 as the first allosteric ligands for the FFA4 receptor. Despite only a minor structural difference between these two compounds, substantial differences in the nature of their allosterism were observed, perhaps suggesting that further examination of the structure activity relationship within the AH-7614 chemical series may yield novel allosteric FFA4 ligands with diverse pharmacological properties. The fact that the observed NAM properties within the AH-7614 series were clearly probe dependent indicates that care must be taken when designing future studies using these ligands. Overall, with appropriate caution, AH-7614 should prove to be a valuable tool in further unraveling functions of the FFA4 receptor.

## Abbreviations

AH-7614: 4-methyl-*N*-9*H*-xanthen-9-yl-benzenesulfonamide
aLA: α linolenic acid
BRET: bioluminescence resonance energy transfer
Cpd A: 3-[2-chloro-5-(trifluoromethoxy)phenyl]-3-azaspiro[5.5]undecane-9-acetic acid
d_6_-DMSO: d_6_-dimethylsulfoxide
DMEM: Dulbecco’s modified Eagle’s medium
ESI-HRMS: electrospray ionization-high-resolution mass spectrometry
eYFP: enhanced yellow fluorescent protein
FBS: fetal bovine serum
FFA1: free fatty acid receptor 1
FFA4: free fatty acid receptor 4
GPCR: G protein–coupled receptor
GSK137647A: 4-methoxy-N-(2,4,6-trimethylphenyl)-benzenesulfonamide
HBSS: Hanks’ balanced salt solution
hFFA1: human FFA1
hFFA4: human FFA4
HPLC: high-performance liquid chromatography
IBMX: 3-isobutyl-1-methylxanthine
IID: 100 nM insulin, 500 mM 3-isobutyl-1-methylxanthine and 10 nM dexamethasone
mFFA4: murine FFA4
mp: melting point
NAM: negative allosteric modulator
pIC_50_: negative logarithm of IC50
PPARγ: peroxisome proliferator activator receptor γ
RT-qPCR: reverse-transcription quantitative polymerase chain reaction
TUG-1197: 2-(3-(pyridin-2-yloxy)phenyl)-2,3-dihydrobenzo[d]isothiazole 1,1-dioxide
TUG-1387: 4-methyl-N-(9H-xanthen-9-yl)benzamide
TUG-1506: 4-methyl-N-(9H-thioxanthen-9-yl)benzenesulfonamide
TUG-770: 4-[2-[2-(cyanomethyl)phenyl]ethynyl]-2-fluorobenzenepropanoic acid
TUG-891: 4-[(4-fluoro-4'-methyl[1,1'-biphenyl]-2-yl)methoxy]-benzenepropanoic acid
